# The Medical Treatment of Insanity

**Published:** 1847-05

**Authors:** 


### ECLECTIC DEPARTMENT,

### AND SPIRIT OF THE MEDICAL PERIODICAL PRESS.

*The Medical Treatment of Insanity.* By A. Brigham, M. D., Superintendent of the State Lunatic Asylum of Utica, N. Y., and Editor of the American Journal of Insanity.

We copy the following from the American Journal of Insanity, April, 1847.—Ed. Buff. Jour.

"We are often questioned by letter and otherwise as to the medical treatment of the insane. To answer some of these inquiries, we think it best to very briefly state our views on this subject, and in a very general manner describe the practice adopted at the New York State Lunatic Asylum.

"No specific remedy for insanity has as yet been discovered.—Different cases require very different treatment, and that which would be serviceable at one period of the complaint might be injurious at another. According to our experience, recent cases for the most part require a mild antiphlogistic course; but regard should be had to the cause of the insanity. If occasioned by a blow, or other direct physical injury to the head, or by some sudden and violent mental commotion, while in good health, free depletion by bleeding, and active cathartics are useful and often indispensable. But such cases are seldom seen in Lunatic Hospitals. We have very rarely considered it advisable to have recourse to general bleeding at this Institution. Only four of the 622 patients that have been here during the past year have been bled by us. In three of these cases the bleeding did not appear to be serviceable; in one we thought it highly beneficial. Occasionally, when there is much cerebral excitement, we have resorted to topical bleeding, but more frequently, even in such cases, we derive benefit from placing the feet in warm water ; the application of cold, to the head ; and the free movement of the bowels by laxatives. Pouring cold water in a small stream from a height of four or five feet directly upon the head, is generally one of the most certain means of subduing violent maniacal excitement, we have ever seen tried. But this should be done in a gentle manner, and under the immediate observation of the physician, and should not be continued but for a short time ; we also advise never to resort to it when the patient's bowels are confined or when he has just been eating and his stomach is full. The warm bath is also serviceable in many cases to calm excitement ; but for this purpose it should be long continued, at least half an hour, and cold water should be gently applied to the head at the same time.

In a few recent cases, croton oil has proved very beneficial, and we have thought particularly so in some cases, that seemed to be cured by the use of it, after some cathartics has been tried. Of all medicines, it is the most easy to administer to a patient that refuses to take any, and we have often used it, and never with an unpleasant result.

Bathing in warm water we think beneficial in most cases. Bathing in cold water or showering, we seldom resort to, probably we should have recourse to the latter more frequently, if not from the impossibility of preventing patients from supposing it to be intended as a punishment.

Most of the medicines we administer are liquid, or in powder.—In addition to the preparations of the articles of the Materia Medica according to the United States Pharmacopœia, we have a few of which we make use, that are prepared by ourselves. The following we often administer :

| | | |
|---|---|---|
| ℞. Extract of Conium, | | oz. vi. |
| Ferri Carb. Precip. | | oz. vii. |
| Molasses, | | |
| Wine, | | |
| Water, (warm) | *a a* | qts. ii. |
| Ol. Gaultheria or Ol. Sassafras; | | dr. ii. |
| dissolved in Alcohol, | | oz. viii. |
| M. | | |

Usual dose half an ounce—to an ounce ; if a laxative effect is wanted, we add one or two drachms of Tinct. Aloes and Myrrh, to each dose.

We sometimes vary the foregoing preparation as regards all the articles except the Conium and iron, adding mucilage Gum Arabic, Alcohol, &c.

The following preparation we derive benefit from in many nervous, sleepless, and hysterical cases.

| | | | |
|---|---|---|---|
| ℞. | Tincture Lupuline, | | |
| | " Hyoscyamus, | *a a* | oz. iv. |
| | Camphor gum, | | dr. i. |
| | Ol. valerian, | m | xxxii. |
| M. | Dose one to two drachms. | | |

The following preparation, we find useful in some cases of violent mania, and when as is often the case, the urinary secretion is deficient.

| | | | |
|---|---|---|---|
| ℞. | Tinct. Digitalis, | | |
| | " Scillae, | *a a* | oz. ss. |
| | Vin. Antimon. Tart. | | |
| | Spts. Nitre dulc. | *a a* | oz. i. |
| M. | Dose 30 to 60 drops. | | |

Blisters, issues, and particularly setons in the neck, we have often tried, but rarely witnessed any benefit from them, unless they sometimes serve to direct the attention of the patient from his imaginary sufferings and delusions, and thus indirectly do some good.

Emetics and Cathartics we do not often prescribe now, as we have seldom known them serviceable, we are, however, careful to avoid a constipated state of the bowels by the use of mild laxatives or special diet.

Opium has always been used at this Institution in the treatment of insanity, and often with great success. In some cases it appears to be useless, and in a few injurious, particularly in those in which the skin is hot and dry, and the pulse full and hard. But such cases are rare. I do not however, think it a remedy that of itself very often cures this disease, but it is a valuable adjuvant to others, and secures a beneficial degree of calmness, that can not be obtained without it. In some cases however, it seems of itself to affect a cure. Of this we can have no doubt after having seen many patients apparently recover while taking it freely, and immediately relapse on its being withheld, and again recover under its use, and finally, after continuing it for a considerable time, and gradually diminishing the dose. recover and remain well for years without it.

We rarely give very large doses, seldom more than one grain of the Sulphate of Morphine or one drachm of Laudanum at a time, usually less. We generally prefer a solution of the Sulphate of Morphine, two grains to an ounce of water, to any other preparation of opium that we have used. We presume the acetate of morphine, is equally good. In some cases Dover's Powder has a better effect than morphine and sometimes laudanum better than either.

I am pleased to find the experience of others in tbe use of opium in insanity has led them to adopt similar views. Pritchard in the first edition of his work on Insanity speaks disparagingly of its use, but in a later work he says, "There are few disorders in which so much benefit is derived from this remedy as in cases of insanity."

Many cases, especially those of some months continuance, require invigorating diet and tonic remedies. The insanity, or rather the causes that produced the insanity, such as grief, anxiety of mind, intemperance, &c., having already debilitated the system, and much caution is necessary not to increase this debility. Hence, although a patient may not exhibit great maniacal excitement, and appear to have prodigious strength, there is usually danger in depleting.

Many of the patients sent to this Institution, have been injured by too much bleeding and depletion before they were committed to our care. Some we think have been rendered incurable by this treatment, and we cannot forbear remarking, that in our opinion, the work of Dr. Rush on the "Disease of the Mind," in which directions are given to bleed copiously in maniacal excitement, has done much harm, and we fear it is still exercising a bad influence, and we hope no future edition will be issued without notes appended to correct the errors into which the distinguished author has fallen, for want of the numerous facts which have been furnished since his time, and which enable us to see the errors of our predecessors.

The various preparations of bark, quinine, and other tonic remedies are here used, but no one preparation is so generally prescribed as the combination of conium and iron above mentioned. and from none have we seemed to derive more benefit. Ale we often administer with advantage.

In many cases of debility and loss of appetite we have found the following preparation quite serviceable.

| | |
|---|---|
| ℞. Tinct. Cinchona Comp. | oz. i. |
| " Gentian, | oz. iii. |
| " Capsici, | dr. ii. |
| Quinine Sulph. | dr ss. |
| Acid Sulph. | m xv. |

M. Dose one drachm in water, or better in ginger tea.

Insanity is often complicated with other diseases and these need attention. Nocturnal emissions not unfrequently occur to the injury of the patient. In such cases we have derived more benefit from *tincture of muriate of iron* in large doses than from any other remedy, and we have tried very many. The insanity of some females seems to be caused and perpetuated by *passive menorrhagia.* It is apt to occur about the time the uterus is losing its functions, and is difficult of cure. We have sometimes derived much benefit from the use of *tincture of muriate of iron*, but more frequently from the tincture of cinnamon, and tincture of aloes combined, from 20 to 30 drops of each.

It should ever be borne in mind that disease in the insane is very apt to be masked,—that serious disease of the lungs or of some of the abdominal viscera may exist, but without being manifested by the usual symptoms, and may therefore be overlooked without careful examination. In other respects not particularized in these remarks, we are not aware that the diseases of the insane require different treatment from those of the sane.

